# Fact or Fiction, It Is Time for a Verdict on Vasculogenic Mimicry?

**DOI:** 10.3389/fonc.2019.00680

**Published:** 2019-08-02

**Authors:** Andrés Valdivia, Gabriel Mingo, Varina Aldana, Mauricio P. Pinto, Marco Ramirez, Claudio Retamal, Alfonso Gonzalez, Francisco Nualart, Alejandro H. Corvalan, Gareth I. Owen

**Affiliations:** ^1^Faculty of Biological Sciences, Pontificia Universidad Católica de Chile, Santiago, Chile; ^2^Faculty of Medicine, Pontificia Universidad Católica de Chile, Santiago, Chile; ^3^Faculty of Medicine, Universidad de Chile, Santiago, Chile; ^4^Faculty of Medicine and Science, Center of Cellular Biology and Biomedicine (CEBICEM), Universidad San Sebastian, Santiago, Chile; ^5^Faculty of Biological Sciences, Universidad de Concepcion, Concepción, Chile; ^6^Advanced Center for Chronic Diseases (ACCDiS), Santiago, Chile; ^7^Millennium Institute on Immunology and Immunotherapy, Santiago, Chile

**Keywords:** vasculogenic mimicry (VM), angiogenesis, endothelial, model, *in vivo–in vitro*

## Abstract

The term vasculogenic mimicry (VM) refers to the capacity of certain cancer cells to form fluid-conducting structures within a tumor in an endothelial cell (EC)-free manner. Ever since its first report by Maniotis in 1999, the existence of VM has been an extremely contentious issue. The overwhelming consensus of the literature suggests that VM is frequently observed in highly aggressive tumors and correlates to lower patient survival. While the presence of VM *in vivo* in animal and patient tumors are claimed upon the strong positive staining for glycoproteins (Periodic Acid Schiff, PAS), it is by no means universally accepted. More controversial still is the existence of an *in vitro* model of VM that principally divides the scientific community. Original reports demonstrated that channels or tubes occur in cancer cell monolayers *in vitro* when cultured in matrigel and that these structures may support fluid movement. However, several years later many papers emerged stating that connections formed between cancer cells grown on matrigel represented VM. We speculate that this became accepted by the cancer research community and now the vast majority of the scientific literature reports both presence and mechanisms of VM based on intercellular connections, not the presence of fluid conducting tubes. In this opinion paper, we call upon evidence from an exhaustive review of the literature and original data to argue that the majority of *in vitro* studies presented as VM do not correspond to this phenomenon. Furthermore, we raise doubts on the validity of concluding the presence of VM in patient samples and animal models based solely on the presence of PAS+ staining. We outline the requirement for new biomarkers of VM and present criteria by which VM should be defined *in vitro* and *in vivo*.

## Introduction

All cells within our bodies require a continuous supply of blood that contains oxygen and nutrients if they are to thrive. In order to ensure this, a subset of cells may synthesize and secrete Vascular Endothelial Growth Factor (VEGF) in response to certain conditions such as low oxygen levels (a condition called hypoxia). Secreted VEGF then mobilizes and activates pre-existent endothelial cells (ECs) that form new blood vessels in a process called angiogenesis. In normal tissues, angiogenesis plays a key role in fetal development and tissue repair. As a consequence, this process is highly conserved among mammals.

As occurs with other physiological processes, cancer cells can hijack angiogenesis in order to potentiate their survival and propagation. Indeed, “tumor angiogenesis” was described 80 years ago and has been extensively confirmed in a variety of experimental models, demonstrating that tumor growth is accompanied by the formation of new blood vessels. Based on these findings, in 1971 Judah Folkman hypothesized that the inhibition of angiogenesis in cancer cells could be therapeutic, coining the term “anti-angiogenesis.” In recent years, several compounds with anti-angiogenic activity have been tested in cancer patients with disparate results; in many cases a favorable initial response is followed by tumor recurrence.

Evolutionary biology teaches us that a selection pressure can generate a resilient system via the natural selection, as such cancer cells (like any other cell) exposed to anti-angiogenic drugs may develop a number of strategies to circumvent the suppression of angiogenesis with these therapies. These strategies include: use of alternative angiogenic pathways, vessel co-option and vasculogenic mimicry (VM) among many others [for a full review see (1)].

In lay terms, VM occurs when a subset of cells within a tumor modify their expression profile/phenotype and form EC-free (i.e., non-angiogenic) tubular structures that supply oxygen and nutrients to cancer cells. Although the existence of VM in tumor samples has been extensively demonstrated there are a number of controversies surrounding published *in vitro* and *in vivo* VM models. Here, we review and discuss the available evidence and controversial issues around VM, seeking to provide a critical assessment of the current literature and a final verdict on the validity of these models.

## What Is Vasculogenic Mimicry (VM)?

As pointed above, the term VM (also reported as vascular mimicry) was originally used to describe the process by which tumor cells formed a network of tubular structures with the ability to conduct fluids, thereby “mimicking” the vasculogenic process of ECs during angiogenesis. Several studies have reported VM both *in vivo* and *in vitro* (see Supplementary Table SII). As explained, the proposed functions of VM are: oxygen supply, nutrient transport, and the elimination of cell waste. These are all critical functions required at the early stages of invasive tumorigenesis that may not be fully accounted by conventional angiogenesis. More recently, the term VM has been expanded to incorporate any EC-free fluid-conducting structure (i.e., not a blood vessel). This came after a study in knockout mice demonstrated that macrophages suffer a phenotypic change acquiring the ability to form fluid-conducting structures (2). Furthermore, prior to the formation of the placenta, trophoblast cells infiltrate the uterine walls, and form EC-free tubular structures that resemble VM (3), suggesting VM may be responsible for blood and nutrient supply in the early stages of pregnancy.

But exactly how can we explain this “phenotypic switch” that allows the formation of vascular structures without ECs? An answer to this question may lie in the vessel structure of *Amphioxus* (*Branchiostoma lanceolatum*), an invertebrate cephalochordate with a body plan similar to that of vertebrates. Like vertebrates, *Amphioxus* vessels are lined by an extracellular matrix (ECM) however the endothelial basement membranes in vertebrates display some differences in their molecular composition (4). These studies not only provide some hints on the evolutionary origins of VM but also demonstrate that EC-free vasculatures are not exclusive to malignancy. In fact, this might be yet another example of an existing or ancient physiological pathway being hijacked by cancer cells.

**Figure 1:**
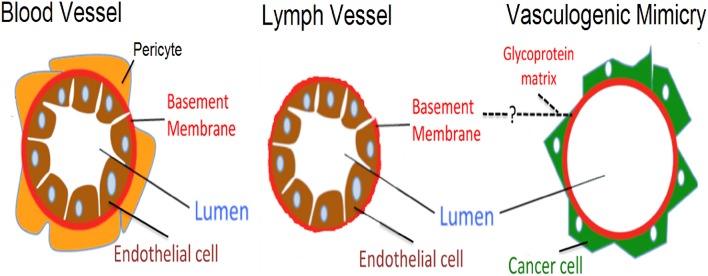
Schematic representation highlighting differences between blood vessels, lymph vessels, and VM structures. Differences are shown between an endothelial lined blood vessel (**left side**), a lymph vessel (**center**), and the hypothesized VM structure (**right side**). Blood vessels present an external layer of pericytes which overlay a basement membrane, with endothelial cells lining the luminal side. In lymph vessels, a similar architecture is present, however pericytes are absent and the basement membrane is thinner. Finally, the proposed structure present in VM has cancer cells sitting on the non-luminal side of a glycoprotein-rich (PAS+) matrix, with the total absence of endothelial cells.

Although angiogenesis, lymph vessel formation and VM share the same goal of establishing fluid-conducting structures within a tissue, they display some notorious differences. Figure 1 shows a comparative diagram of traditional blood vessels (formed by vasculogenesis or angiogenesis), lymph vessels, and VM vessels. In traditional blood vessels (left), a single layer of ECs lines the lumen: an external continuous inner-basement membrane surrounds ECs in these vessels. Similarly, lymph vessels have a central inner layer of ECs; however, their basement membrane is non-continuous (Figure 1 center) (5). Our current understanding of VM vessels suggests that cancer cells sit on top of a glycoprotein rich membrane (matrix) which surrounds a central lumen (Figure 1 righthand panel) (6). As observed in the basement membrane on traditional blood vessels, these studies suggest that VM vessels also have a glycoprotein-rich inner coating composed by collagens and laminin, among other proteins (7, 8). In summary, traditional (or conventional) blood vessels and VM vessels can be identified and distinguished based on structural and composition differences as indicated in Figure 1. These features have been systematically used in the literature to identify VM in cancer patient samples.

## VM in the Clinic: What Is the Evidence in Cancer Patients?

Since its first publication, many authors have embraced the existence of VM, while others have disputed it. The latter argue this is only a remote phenomenon that occurs within tumors and may be open to misinterpretation (9–11). The basal membranes of both blood and lymph vessels contain a variety of mucinous proteins (glycoproteins) that stain positive for the Periodic Acid–Schiff (PAS, mucosubstance stain) (5). Throughout the literature, the existence of VM vessels is inferred by the presence of PAS+ vessel–like structures within tumors in the absence of EC markers such CD34 and CD31, among others. Hence, authors have postulated VM as an angiogenesis-independent alternate tumor perfusion pathway for tumors. Indeed, human tumor biopsies have shown the presence of red blood cell (RBC) containing PAS+ vessels that stain negative for EC markers.

**Figure 2:**
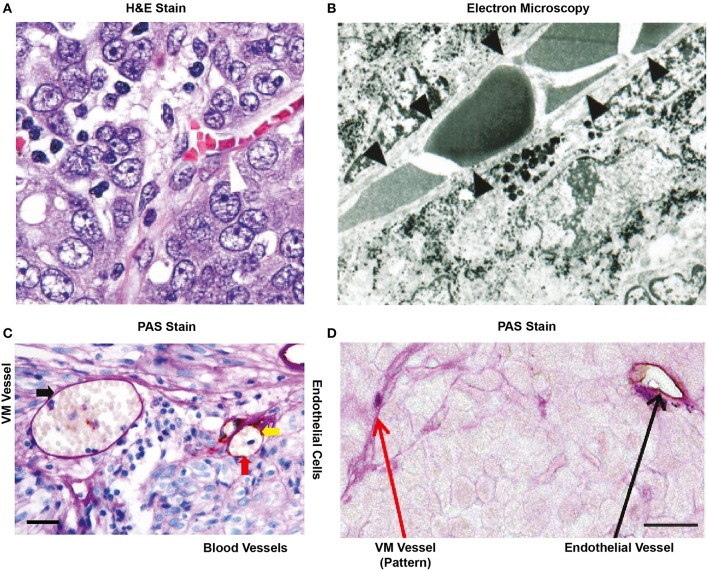
Historical representation of *in vivo* VM from the literature. **(A)** H&E stain of an ovarian tumor showing that red blood cells are contained in a structure lined by cancer cells (arrow head), not endothelial cells. Image taken with kind permission from the publication by Sood et al. (12). **(B)** Electron microscopy of a uveal melanoma showing that red blood cells are contained within a structure lined by cancer cells (as confirmed by the presence of melanin in these cells). Taken with kind permission from the publication by Maniotis et al. (13). **(C)** Cross section of a glioblastoma after staining for PAS and CD31. The red arrow indicates an endothelial lined blood vessel with PAS+ luminal stain and positive for the endothelial marker CD31 (yellow arrow). RBCs (red arrow) are shown within the structure. In the same cross-section, a PAS+ yet, and CD31 negative structure is also present that contains red blood cells (black arrow). This may represent VM. Size bar represents 100 μm. Taken with kind permission from the publication by Sun et al. (14). **(D)** Small lung cell carcinoma cross section showing the presence of a PAS+/CD31+ blood vessel (black arrow) and the presence of PAS+ “patterned structures” that have been speculated to represent VM (red arrow). Size bar represents 100 μm. Image taken with kind permission from the publication by Williamson et al. (15) http://creativecommons.org/licenses/by/4.0/. All appropriate permissions have been obtained from the copyright holders.

Originally described in uveal melanoma, VM is now reported in >20 malignancies (Supplementary Table SI). VM critics such as Professor McDonald have claimed that this is nothing more than an “artifact” consequence of the erratic structure of the tumor endothelium and the accumulation of blood, derived from microhaemorrhages (11). Indeed, this has been a recurrent argument among critics claiming that these structures are merely “blood pools” brought about by the process of tissue acquirement (see Figure 2A) (12). While plausible, this argument does not take into consideration that a trained pathologist can easily distinguish a “blood pool” from RBCs trapped within a tubular structure. Moreover, if these were indeed blood pools, then RBCs would not be enclosed within a PAS+ structure. As an example, Figure 2B shows RBCs surrounded by melanoma cells (black spots are melanin) with black arrows indicating a continuous covering of a tubular structure; this may be interpreted as a basal membrane. However, the field of VM may have itself to blame for the current controversy as several inconsistencies among VM reports have generated skepticism. For example, some studies postulate the presence of VM based on a luminal space in a carcinoma cross-section, however no PAS+ border is present (16). Similarly, weak PAS staining always leaves the doubt of whether a membrane is present or the structure is in fact a blood pool (17–20). In contrast, several reports from the group of Sun and colleagues clearly demonstrate the presence of PAS+/CD31- structures that contain RBCs in both Hepatocarcinoma and Gastro Intestinal Stromal Tumor (GIST) patients (14, 21). Encouragingly and as a proof of concept, these reports demonstrate the presence of both VM (PAS+/CD31–) and blood vessels (PAS+/CD31+) within the same field (shown in Figure 2C). Similar evidence is reported in uveal melanoma, where a fluorescent dye was injected into the patient and tracked through to the eye (13). An exhaustive analysis of glioblastoma by Scully and colleagues showed the presence of CD31, CD34, and/or Vascular Endothelial (VE)-Cadherin+ positive (and thus endothelial) and negative (potentially VM) luminal structures. This study also demonstrated that endothelial confirmed vessels presented alpha smooth muscle actin (SMA, a pericyte marker) while potential VM structures did not (22). Taken collectively these publications demonstrate the existence of non-endothelial blood containing vessels in human tumors.

## Is the Combination of PAS+ and Absence of EC Markers a Definitive Proof of VM?

Not exactly, we agree that the confirmed observation of a PAS+/CD31– lumen containing RBCs maybe indicative of VM. However, we believe this is not a definitive proof. Figure 2D shows thread-like PAS+ structures commonly reported throughout the literature as “patterned structures” (13, 23, 24). As we will describe later, cancer cells secrete large amounts of mucoproteins that stain PAS+, however this does not imply these are forming tubular structures. Another example of patterned structures is shown in Figure 2C; where strands of PAS+ structures can be observed over a “true” blood vessel (RBC containing CD31+ tubular structure). Furthermore, PAS+ “patterned structures” have also been reported in medulloblastoma where potential VM structures are suggested to connect to the EC vasculature (25). However, electron microscopy by Maniotis et al. of pattern structures does suggest that blood components can be present in the vessel interior (26). Thus, the jury is still out on whether all “pattern structures” can be classified as VM. A further problem in the reporting of the presence of VM occurs when no imagery is shown; without physical evidence it is difficult to draw conclusions (27). Similarly, small images in black and white do not allow the reader to be convinced of the presence of PAS+/CD31– structures (28). While these publications may be validly reporting the presence of VM, without a standardized method of reporting this phenomenon it is difficult to verify any conclusion on incidence and function.

In summary, in the absence of a reliable VM biomarker the combination of PAS+ and absence of classic EC markers like Von Willebrand factor, CD34 or CD31, plus RBCs in a clearly defined lumen should be the standard for reporting VM+ status across the literature.

## Why Should We Care About VM?

**Figure 3:**
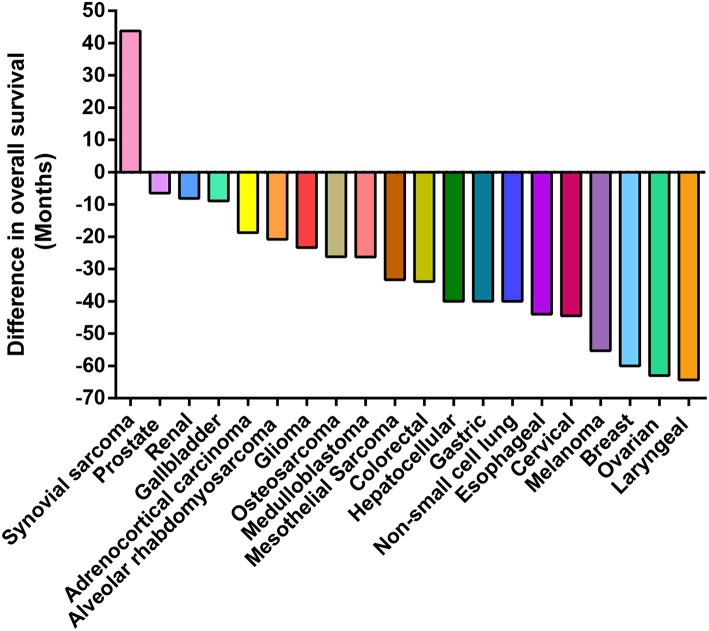
Presence of VM is associated with poor patient prognosis. Waterfall plot presenting the difference in survival time (years) of patients presenting structures claimed to represent VM in their tumors. The zero value represents patient survival in the absence of VM. In reports to date VM has a negative impact on the overall patient survival in all but one cohort (synovial sarcoma).

Because a large number of studies indicate that VM+ is associated to a decrease in cancer patient survival, measured as OS or as progression-free survival (PFS) (13). Supplementary Table SI and Figure 3 compare OS levels in VM+ vs. VM- tumors across 20 cancer types. Overall, 19 out of 20 reports confirm that VM+ associates with a decrease in OS; with the exception in synovial sarcoma (29). Supplementary Table SI summarizes all current literature reporting occurrence rates and OS in pathology observed cancers. Strikingly, reports in ovarian and colorectal cancers classified as VM+ showed lower survival time in the magnitude of years compared to VM- tumors (30, 31). Similar differences were observed in orbital rhabdomyosarcoma and adrenocorticoid carcinomas (32, 33). Gastric cancer patients with PAS+ structures were prone to present higher histological grade, metastasis, distant recurrence, and 12 months less cumulative OS (34). Similarly, VM+ prostate cancer patients correlated with Gleason score, preoperative prostate-specific antigen (PSA) levels, pathological stage and both lymph node and distant metastasis. Studies to date have come principally from the Chinese population, although isolated reports have been published from European, Japanese, North American, and Thai populations. However, as observed in cancer incidence, the frequencies of cancer type and the mutational burden within each classification vary according to region and further studies need to be performed to get a clearer picture of prognostic value of VM presence within a specific population. In summary, the overwhelming consensus of the literature suggests that VM is frequently observed in highly aggressive tumors and correlates with poor prognosis. Therefore, the elucidation of specific treatments targeting this subset of aggressive cells may have offer a benefit for cancer patients in terms of survival.

## Is Vasculogenic Mimicry a “Hallmark of Cancer”?

Or in other words do all cancer cells undergo VM? A short answer to this question would be no. Reports indicate the percentage of VM+ tumors (by PAS+/CD31–) varies wildly from 5 to 65% depending on the cancer type and the pathologists' inclusion criteria. Among the studies that assessed tumor-based data the average VM incidence is about 29%. As shown in Supplementary Table SI, glioblastoma has the highest incidence among tumor types (65.9%), with the lowest incidence to date reported in melanoma (5.25%) (29). There is a notable heterogeneity in the reporting of VM as demonstrated in glioblastoma patients. Han et al. reported a 65.9% of VM+ in glioblastoma patient samples (35), however two similar studies reported 26% (36) and 16% (24) also in glioblastoma. Evidently, patient and/or tumor characteristics such as tumor stage or histological grade, could be responsible, however, to differences in the reporting criteria for VM+ could also be attributed, illustrating the need for a standard classification.

## The *in vivo* Controversy: An Animal Model of VM

As in all *in vivo* models of cancer the mouse xenograft has been the standard for VM research. Initial studies of breast cancer cell xenografts were assessed for VM by Hematoxylin & Eosin (H&E) stain and investigators acknowledge that by using this technique alone, a pathologist could misinterpret VM as blood pools caused by internal tumor hemorrhages (37, 38). Later the same year utilizing LnCaP prostate cell xenografts stained by H&E and prostate specific membrane antigen (PSMA) demonstrated structures that were CD31– yet positive for platelet aggregates and fibrin (39). The first report that used the PAS+/CD34– combination came in a model of B16 melanoma cells injected into C57Bl/6 mice (40). This pioneering study demonstrated the presence of PAS+ non-EC structures that contained RBCs within their lumen (40). Following this study, several authors reported PAS+/CD31+ (blood vessel) or PAS+/CD34– (VM) structures, however, in some cases low quality or low-resolution images failed to prove CD31– status or presence of RBCs (37, 41–43). In contrast, a number of studies have provided solid evidence of PAS+/CD31– stained structures that also contain RBCs in their lumen (29, 39, 44–46).

The current tools to identify VM *in vivo* are clearly deficient! PAS+ staining alone does not guarantee VM presence and thus novel biomarkers that discriminate between VM and blood pools are urgently needed. As potential biomarkers, Bajesy et al. used 3D Z-stack reconstructions to identify intratumoral structures that were both laminin+ and CD34- in metastatic uveal melanoma samples (47). A recent study used a pan-laminin antibody along with an EC-binding lectin to identify VM structures in xenografted human glioblastoma cells (48). The authors demonstrate the presence of lectin+ and lectin- tubular structures. These results suggest the mucoprotein content and composition of these tubular structures may vary substantially. Hence, future studies could aim to identify specific mucoproteins within CD31- vessels, perhaps specific lectins or other ECM components that will improve current VM identification methods.

## The *in vitro* Controversy: the Principle Problem

The presence of an *in vitro* model is potentially the most controversial aspect of the VM field. To understand this fully and to trace the errors that have occurred within our scientific discipline, in the following paragraph we examine the origin of the *in vitro* model and speculate how the majority of the papers in medical literature may be erroneously presenting conclusions based on an assay that is not measuring VM.

During the 2001–2002 period Mary Hendrix's group published several articles providing the first evidence suggesting that VM structures contained a lumen, lined by a glycoprotein-rich membrane (12, 49–55). This process only occurred in a 3D matrix (Matrigel) and after several days in culture. A study by Sanz et al. (56) was the first to present an *in vitro* assay claiming that intercellular connections formed within 1 day of cancer cell culture in Matrigel represented VM. These structures initially were thicker that those observed in the classic tube forming assays using endothelial cells (classic angiogenesis assay using HUVECs or EC lines) however, this study failed to proof these were functional lumen containing structures (i.e., could conduct fluid). Furthermore, the study proposed a quantification method based on cellular connections (56). This could have been a turning point in VM research, as these structures (and structures which were slightly thinner and more similar to those seen in angiogenesis assays) became adopted as an accepted *in vitro* representation of VM. A subsequent study by Vartanyan et al. described side by side EC and cancer structures claiming that both were lumen containing and that the VM was a representation *in vitro* of the blood filled CD31- vessels seen in histological cross-sections of tumors (57). Perhaps the greatest contributor to the current controversy came in 2011 when Francescone et al. published a paper entitled “*A Matrigel-based tube formation assay to assess the vasculogenic activity of tumor cells*.” This has been cited as a reference validating the concept that intercellular connections represent VM ever since (58). Although there have been notable exceptions, most of the VM research *in vitro* has utilized intercellular connections formed between cancer cells to report the presence and mechanisms of this phenomenon. Thus, the field of VM, at least *in vitro*, has continued to be shrouded in controversy, leading to divided opinions in the scientific community.

## Back to Basics: The Hendrix Model Revisited

Initial representations shown by the Hendrix group of VM *in vitro* demonstrated tubular structures that formed after numerous days in culture, that where lumen containing and importantly were capable of fluid conduction (6). Herein, we suggest that this model, with improvements, should be the standard for *in vitro* assays of VM.

**Figure 4:**
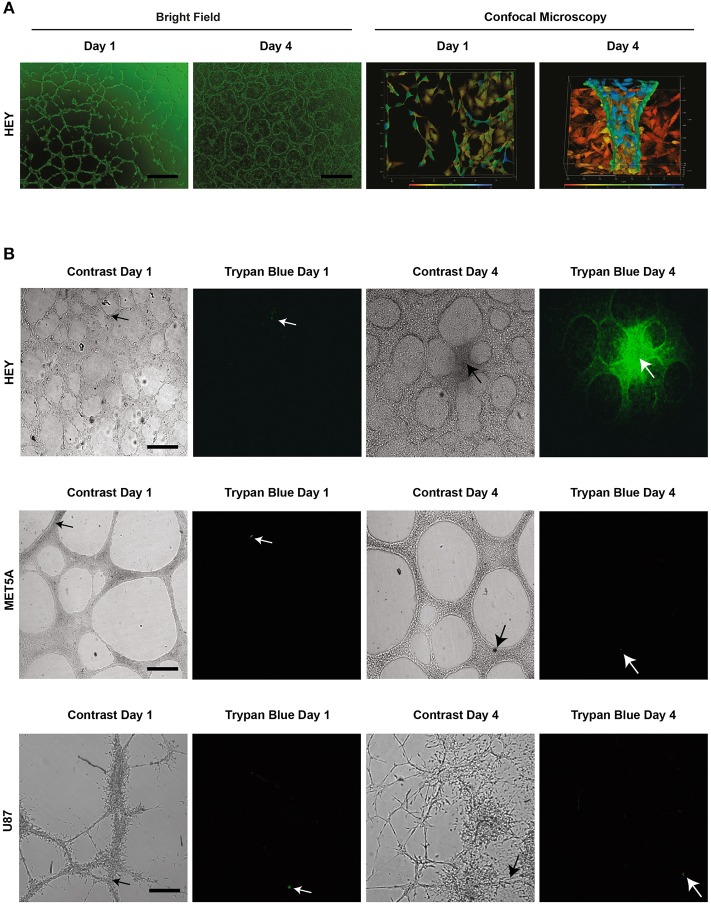
Structures present in matrigel culture may not represent VM. The upper panel **(A)** demonstrates light (size bar represents 200 μm) and confocal microscopy (size bar represents 500 μm) images of day 1 and day 4 cell cultures of the HEY cancer cell line. The presence of a tubular hollow structure can clearly be seen at day 4 in culture. **(B)** demonstrates dye microinjections of the HEY cell lines at day 1 and 4, showing movement of fluid and thus functional tubes (upper panel). Despite the formation of what is often reported in the literature to refer to tubular structures (VM), no movement of dye was observed in the MET5A or the U87 cell lines at day 1 and 4 (middle and lower panel respectably). In fact, the dye either remained in an individual cell or dispersed from culture. Arrow heads display injection sites, all size bars represent 250 μm.

To elaborate upon this idea and to demonstrate to the reader that intercellular connections or a congregation of cells do not represent fluid containing vessels, Figure 4 depicts representative imagery complementing previous results presented by our group and in line with the initial representations shown by the Hendrix (6). In this figure there are two cell lines that demonstrate structures reported to be VM in the literature. Figure 4A shows the HEY cancer cell line forming intercellular connections at day 1 in Matrigel culture, which become develop into elevated structures above a cell monolayer at day 4. However, the appearance of intercellular connections on the first day does not necessarily mean that VM structures will occur at a later date. With the aim of demonstrating cell lines that form intercellular connections but do not produce a hollow lumen or conduct fluid, Figure 4B shows the formation of network structures at day 1 and 4 in the MeT5A and U87 cell lines. We observed that if we inject a fluorescent dye into 1 and 4 day-old structures there is no dye movement; the dye stays diffusely only around the individual cell that receives the injection (actually it almost below detectable levels, hence the black image). However, dye movement is observed in Day 4 cultures of the HEY cell line. As was shown in a previous publication, injecting the dye into individual cells of the HEY cell monolayer does not result in dye movement and furthermore, injecting dye into structures spanning clusters of cells in other cancer cell lines (UCI101 and A2780) also fails to show presence of a fluid conducting tube [this can be observed in Figure 4c of (6)].

These results suggest that although there appear to be tubular structures, only intercellular connections or cellular aggregates are present, and thus the majority of structures presented as VM in the literature may not in fact contain a lumen and are thus incapable of fluid conduction. Although an argument could be made that some of the published intercellular structures shown at day 1 may develop into VM tubular structures, the authors cannot be sure of this claim and thus we suggest that the model is not valid. In our own work on primary cultures we often saw initial intercellular connections during the first day in culture that subsequently disappear after several days (6). Following this line of thinking, a future area of controversy may be the report of intercellular connections formed after 12–24 h in Matrigel of cell lines at that have been previously reported to produce fluid conducting structures at latter time points. While this may be currently acceptable, it is dangerous to assume that anything that inhibits tubular structures at day 1 is specific to the pathway required for the process of VM. Any tested compound or pathway component may in fact be representing toxicity to the cell, an inhibition of cell cycle or a change in cytoskeleton that will inhibit all movement related biological processes such as migration and invasion. We recommend that assays examining the process of VM be followed to the formation of undeniable fluid conducting structures.

In Supplementary Table S1 we have divided the publications in the field of VM into those that either demonstrate or fail to show the presence of a lumen and/or conduction of fluid. This analysis reveals that of the 357 published papers reporting VM *in vitro*, only 49 (13.7%) convincing demonstrate a tubular structure. Although, this does not mean that all reports of tubular structures within the first 24 h (intercellular connections) will not eventually form VM structures, it is impossible based on this assay to distinguish between merely intercellular connections or the process of VM with the presence of fluid conducting tubes. A universally accepted model of VM that demonstrates a lumen or fluid conduction is required for research in this field to advance. Furthermore, conclusions based on assays that do meet these criteria should be interpreted skeptically.

## Presentation of a Standardized *in vitro* Model of Cancer VM

**Figure 5:**
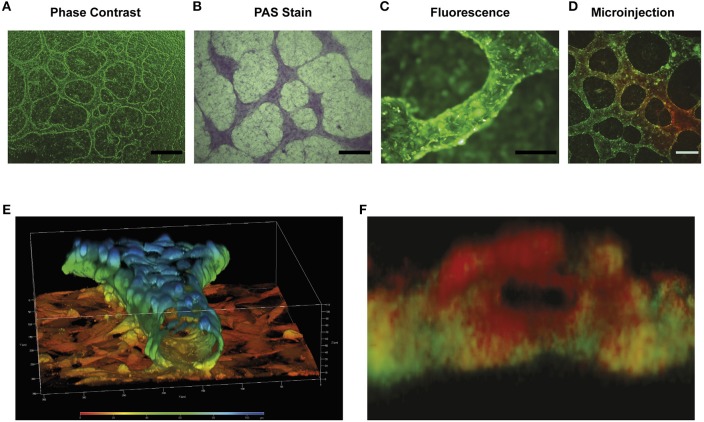
Characterization of *in vitro* model of VM: **(A)** is a representative light microscope image of the tubular structures formed by the HEY ovarian cell line after 4 days in matrigel culture. Elevated tubular structures are observed above a cell monolayer. Size bar represents 500 μm. **(B)** PAS stain showing that the elevated tubular structures possess a higher concentration of glycoproteins that cell monolayer present below. Size bar represents 500 μm. **(C)** Epifluorescence image of the tubular structures of the formed in the HEY cell line transfected with GFP (HEY-GFP). Note the difference in height of the tubular structure compared with cell monolayer below and that the tubular structure (~100 μm in diameter) is composed of numerous cancer cells. Size bar represents 200 μm. **(D)** A dye micro injection of 4-day old HEY-GFP culture, showing that these structures are capable of moving a colorant dye within their interior. Size bar represents 200 μm. **(E)** Confocal 3D reconstruction of the tubular structures found in the HEY-GFP cell lines using ZEN 2012 program. The color scale represents the height in culture over the cell monolayer. Note how the cells form a tubular hollow structure with a central lumen. The size bar demonstrates that the structure is ~80 μm in diameter. **(F)** A 3D reconstruction of a cross section of the tubular structure showing the PAS positive (red) show up preferentially on the luminal side of the tubular structure, while the green cancer cells are confined to the outer sides (as schematically represented in Figure 1). Size bar represents 100 μm.

In our opinion only a few *in vitro* studies have convincingly demonstrated a functional lumen in tubular structures (12, 49–55). Building upon these pioneering studies, our research group recently published an *in vitro* model demonstrating (we believe convincingly) that cancer cells grown in Matrigel form tubular structures with a central lumen lined by glycoprotein-rich borders (Figure 5). After several days in culture, cancer cells originate PAS+ structures that appear to be atop cancer cell monolayers. These PAS+ structures may reach up to 200 μm in diameter (Figures 5A–C). The movement of microinjected trypan blue dye along these structures confirms they contain a functional lumen (Figure 5D). Confocal microscopy and IMARIS (Microscopy Image Analysis Software) reconstruction further confirm the presence of a lumen and a glycoprotein-rich layer flanked by cancer cells (see VM vessels in Figure 1). Our data indicate these structures can be obtained in Matrigel cultures derived from cancer cell lines, primary tumors or from patient ascites (6). In 13 advanced ovarian cancer patient samples, only 38.5% (5 out of 13) of samples were capable of producing tubular structures *in vitro*. Previous studies report that 29–43% of ovarian cancers samples analyzed by immunohistochemistry present PAS+ and endothelial marker negative structures, thus we speculate that the ability of a tumor cell population to undergo VM may be retained *in vitro* (59).

## VM Quantification: Is PAS a Good Marker?

No, as we explained above PAS+ along with absence of EC markers allows VM identification but it is not sensitive enough to allow quantification. The literature on *in vitro* VM models contains several attempts for a quantification method. Such studies have employed a variety of methods including: tubule length, number of structures, tubular structure connections, or PAS+ levels (16, 17, 60–62). However, as explained above most studies have failed to demonstrate these tubular structures are indeed functional (i.e., have a fluid-conducting lumen) therefore the validity of these methods remains questionable.

**Figure 6:**
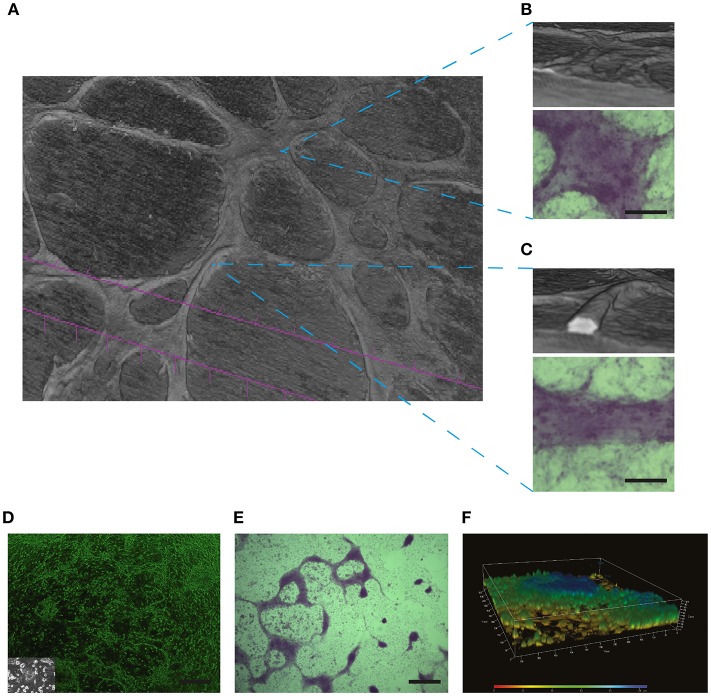
PAS positive structures may not necessarily represent the presence of VM: In **(A)** Micro-CT 3D reconstruction of the SKOV3 ovarian cancer cell line after 4 days culture in Matrigel demonstrating the presence of elevated structures over a cell monlayer. **(B)** A flatter yet elevated section of the culture did not demonstrate a lumen when reconstructed by Micro-CT, yet this structure stained positive for PAS (lower panel). Size bar represents 100 μm. **(C)** Reconstruction by Micro-CT demonstrates the presence of a lumen containing structure, as demonstrated by the interior white space. The PAS positive stain is shown in the lower panel. Size bar represents 100 μm. In **(D–F)** a characterization is shown of structures formed by primary cultures of glioblastoma cells. These samples where obtained with Ethical Committee approval and written patient consent from the Clinical Hospital of the University of Chile, Santiago, Chile. Cell culture was as described previously in Racordon et al. (6). **(D)** Light microscopy imagery of primary cultured cells grown on matrigel, with an image of the cells grown in plastic in the inlay. Size bar represents 500 μm. **(E)** Primary cultured glioblastoma cells presented elevated structures over the cell monolayer that stained for PAS. Size bar represents 500 μm. **(F)** Confocal 3D reconstruction using ZEN 2012 demonstrates that the PAS positive structures observed in **(E)** are elevated over the cell monolayer but do not possess a lumen.

Historically, PAS has been used as a staining method to identify mucosubstances such as glycoproteins, polysaccharides, and glycolipids (63). While VM channels clearly display a strong PAS+ stain (6, 13, 64), our micro-CT analyses [shown in Figure 6 and also in Supplementary Video 1 and Figure 2 of Racordon et al. (6)] demonstrate that in many cases PAS+ structures do not contain a lumen. Using this micro-CT technique, we observe tubular-like structures along with flatter areas that also stain heavily for glycoproteins (PAS+). In this technique, white areas denote air-containing structures. In Figure 6B we can observe that the flatter less tubular elevated structures do not possess a hollow structure. Alternatively, the Figure 6C, demonstrate rounded structures that clearly contain a lumen (white area). Hence, PAS staining in some cases may just represent glycoprotein-rich areas around aggregations of cancer cells. Accordingly, PAS+ structures obtained on a glioblastoma cell culture in culture (Figure 6D) are not able to conduct fluids. The lack of a lumen in these structures is further confirmed by confocal microscopy reconstruction (Figure 6F).

Thus, as we move toward a standardization of non-endothelial vessels/VM, until a unique biomarker has been identified, the use of PAS+ staining alone should be viewed cautiously and the reporting of “pattern structures” (shown in Figure 2D) should be replaced by PAS+ straining accompanied by the absence of an EC marker and preferably the presence of RBCs in a luminal structure.

## In Search of the Signaling Pathway Leading to VM Formation

Beyond the controversy over the nature, definition, and identification of non-endothelial vascular structures, a number of articles have sought to define a mechanism for tubule formation. Most studies used an *in vivo* approach, double stain PAS+/CD31– or PAS+/CD34– for VM+ and then correlated these structures with molecular markers (12, 65–68). Other studies have used pharmacological inhibitors on *in vitro* models (66, 69, 70). Using our criteria for true VM structures: PAS+/CD31– or PAS+/CD34– and presence of a lumen for *in vivo* and *in vitro* studies we elaborated a list of 93 articles that fulfilled these criteria and also postulated a VM mechanism based on molecular pathways (Supplementary Table SIII). We found that signaling/molecular pathways across all relevant literature could be grouped into 4 specific areas:

### Matrix Metalloproteases and Extracellular Matrix Components

A number of reports have suggested a role of matrix metalloproteases (MMPs) in VM. Sood et al., were the first to demonstrate a correlation between VM+ and expression of metalloproteases (MMPs)-1, MMP-2, MMP-9, MMP-14 in ovarian cancer samples (12). These studies also reported an association with Laminin-5 ⋎-2. T ECM rearrangements and the secretion or incorporation of laminin subunits. A subsequent report showed that MMPs and Laminin-5 ⋎-2 were required for the formation of VM in melanoma (51). In prostate cancer, VM+ correlated with laminin and integrin α6β1 (52) and in mesothelial sarcomas and alveolar rhabdomyosarcomas with the presence of collagen IV fibers (29). In 2008, Demou reported that VM+ was associated to the presence of integrin α3 subunit (71). As it is established that *in vitro* VM only occurs upon an ECM substitute (Matrigel), it may be reasonable to assume the process requires ECM remodeling by MMPs. Future experiments will need to elucidate whether ECM is the source of the glycoprotein-rich lined lumen observed in tubular structures *in vitro* or if this glycoprotein is secreted by the cancer cells.

### PI3K-AKT Pathway

A study by Hess et al. (72) was the first of several studies to implicate the phosphoInositide-3 kinase (PI3K)-AKT pathway in VM (72–74). Subsequently, the same research group presented evidence for a role of focal adhesion kinase (FAK), an upstream component of the PI3K pathway and important component of the integrin signaling pathway (75). Two related studies demonstrated VM structures were associated to AKT (76) or correlated to MMPs, PI3K and FAK (68) in melanoma and gallbladder cancer, respectively, adding to the possibility that the integrin-FAK and PI3K-AKT signaling pathway are also involved. In our opinion, the PI3K pathway has provided the most solid evidence to date for a role in VM formation; this could also offer the opportunity for a therapeutic intervention in the future.

### Angiogenesis Signaling Pathways

As both VM and angiogenesis result in tubular fluid-conducting structures, it would appear logical that they have signaling pathways in common. However, the relationship between VM and angiogenesis is a controversial topic. Many authors have reported that the angiogenesis signaling pathway plays a role in VM, with a correlation between VM+ and either VEGF or PDGFRβ expression in cancer samples (22, 37, 54). Another factor associated to VM is the Hypoxia Inducible Factor (HIF)-1α, its presence is also widely linked to the stimulation of pro-angiogenic pathways (65, 77–80). However, in sharp contrast, some reports demonstrate that antiangiogenic therapies, such as treatments against VEGF or its receptors have no impact upon VM, demonstrating the inconsistencies across the VM literature (48, 81, 82). Indeed, several studies speculate VM is a key process that allows tumor irrigation and growth even in the presence of anti-angiogenic therapy (1, 68, 83). Evidently, the lack of a consensus on the criteria to report VM may explain why the role of proangiogenic factors on VM remains unclear.

### Other Signaling Pathways

Complementing the abovementioned studies, further reports have speculated on key components of VM formation. VM presence and poor patient prognosis has been reported with Tissue Factor Pathway Inhibitor-1 (TFPI-1) and TFPI-2. Antibody inhibition experiments revealed that TFPI-2 was required for VM *in vitro*, and that the blockade of TFPI-2 suppressed MMP2 activation (41). Whether this suggests that the coagulation cascade is involved in VM, or a non-homeostatic role of these proteins is responsible, has still to be evaluated.

Given the presence of fluid conducting tubular structures, VE-Cadherin has also been commonly associated to VM (84). VE-Cadherin is a cell-adhesion transmembrane protein classically expressed in ECs (85). Hendrix et al. described the presence of VE-Cadherin in melanoma cells undergoing VM (86). Furthermore, in melanoma VE-Cadherin has been reported to promote VEGFR-1 signaling, that in turn promotes the signaling of the PI3K/PKC pathway, which is critical for VM (87, 88). However, despite isolated reports it is still open for investigation to determine whether the process of VM is using similar pathways to that of angiogenesis or vasculogenesis.

The Wnt signaling pathway and EMT, commonly implicated in cancer, angiogenesis, and development have also been implicated in VM formation (89, 90). Wnt3a and β-Catenin are shown to increase formation of tubular structures in colon cancer (91), while essential EMT proteins Slug, Snail, and Twist, have been correlated with the presence of tubular structures (92, 93). While it may appear logical that developmental signaling process and pathways would be implicated in VM formation, the abovementioned publications, together with numerous others, further demonstrate that the true mechanism of VM formation is still to be defined.

## Concluding Remarks and Future Directions

It was 1971 when Judah Folkman first postulated that the inhibition of tumor angiogenesis could be therapeutic, coining the term “anti-angiogenesis” to refer to the suppression of tumor blood supply (94). At the time, the rationale behind tumor irrigation seemed quite simple. However, over time we have learned that cancer cells (like any cell) have the ability to adapt and evade treatment regimens by systematically activating pathways and tools already present within our genome to ensure continuous self-propagation. Indeed, cancer cells can develop a number of strategies to compensate for angiogenesis and/or circumnavigate the inhibition of specific angiogenesis pathway by using alternative/compensatory pathways, vessel co-option or VM (1). We speculate that VM plays a key role in both bourgeoning tumors and in the evasion of antiangiogenic treatments. A standardization of assays for VM detection and quantitation in clinical samples along with reliable *in vitro* VM models will allow the development of biomarkers, drug discovery, and more effective treatments for antiangiogenic refractory patients.

Regarding VM biomarkers, the evidence suggest PAS alone may not serve as an effective biomarker (6). Novel, more specific biomarkers are required to discriminate endothelial vs. non-endothelial structures. Furthermore, it is critical to determine if “pattern structures” represent structures with a true lumen or merely polls of glycoproteins secreted by tumor cells. For now, we suggest pathology-based VM reports should demonstrate: PAS+, absence of EC markers, and a lumen containing RBCs.

Regarding the elucidation of a VM mechanism the interpretation of the literature is arbitrary, at best. In our opinion, most studies that provide a VM mechanism of action are based on *in vitro* assays that unfortunately need to be discarded, or at best treated with skepticism. To date, mechanistic data have come almost exclusively from *in vitro* models that wrongfully interpret intercellular connections as formation of VM and therefore should be assessed with caution. On the other hand, VM studies based on immunohistochemistry of tumor sections cannot deliver mechanisms, only association for example enrichment of EMT-related proteins or HIF-1α expression (65, 77, 78). Studies to date have failed to provide a gain-of-function/loss-of-function system for VM either by chemical inhibition or gene silencing.

In summary, reliable *in vitro* and *in vivo* VM models are urgently required and need to be universally adopted by the scientific community in order to identify, quantitate, and elucidate the mechanisms behind this phenomenon. The delivery of a clinical marker for VM could serve as a marker for anti-angiogenic treatment refractory patients. Finally, reliable VM models may identify actionable targets and thus finally accomplishing Judah Folkman's dream of total suppression of tumor irrigation.

## Data Availability

All datasets generated for this study are included in the manuscript and/or the Supplementary Files.

## Ethics Statement

This study was carried out in accordance within the guidelines and recommendations of the Ethics and Bioethics committees of the Servicio de Salud Metropolitana Oriente (15122015) and the Pontificia Universidad Catolica de Chile (resolution 13-226, FONDECYT 1180241, 2018). All subjects gave written informed consent in accordance with the Declaration of Helsinki.

## Author Contributions

AV, GM, VA, MR, CR, and FN performed literature searches, experiments, and assisted in preparing the manuscript and figures. MP, AG, AC, and GO designed and wrote the manuscript.

### Conflict of Interest Statement

The authors declare that the research was conducted in the absence of any commercial or financial relationships that could be construed as a potential conflict of interest.

## Supplementary Material

Supplementary Table SI Presence of vasculogenic mimicry and cancer patient survival. Papers presenting overall survival cancer patient survival were used for analysis. Patient survival was converted to months to unify the data.

Supplementary Table SII Vasculogenic mimicry in the literature. *In vivo* model: Y = Yes; N = No. *In vitro* model: Y = Yes; N = No. *In vivo* characterization: 1 = H&E/PAS only; 2 = H&E/PAS-CD31; 3 = H&E/PAS-CD34; 4 = Other (leptin marker, laminin marker, electron microscopy, etc); A = Lumen containing structure only; B = Lumen containing structure with red blood cells; C = VM Pattern (PAS Stain). *In vitro* characterization: 5 = only light microscopy; 6 = light microscopy/PAS; 7 = electron/confocal microscopy; 8 = lumen presence demonstration; 9 = functionality demonstration (microinjection); W = Others; X = cells projection; Y = tubular structures; Z = Not Shown. Xenograft model: Y = Yes; N = No.

Supplementary Table SIII Summary of the literature presenting mechanisms of action for vasculogenic mimicry. To be considered to represent a valid mechanism of VM formation the publications had to include the criteria of *in vivo* and/or *in vitro* characterization of 2-A, 3-A, 4-A, 2-B, 3-B or 4-B; or 5-Y, 6-Y, 7-Y, 8-Y, 9-Y or W-Y. *In vivo* model: Y = Yes; N = No. *In vitro* model: Y = Yes; N = No. *In vivo* characterization: 1 = H&E/PAS only; 2 = H&E/PAS-CD31; 3 = H&E/PAS-CD34; 4 = Other (leptin marker, laminin marker, electron microscopy, etc); A = Lumen containing structure only; B = Lumen containing structure with red blood cells; C = VM Pattern (PAS Stain). *In vitro* characterization: 5 = only light microscopy; 6 = light microscopy/PAS; 7 = electron/confocal microscopy; 8 = lumen presence demonstration; 9 = functionality demonstration (microinjection); W = Others; X = cells projection; Y = tubular structures; Z = Not Shown. Xenograft model: Y = Yes; N = No.

Supplementary Video 1 Dye Microinjection of Different Structures Formed by the U87 and Hey Cell Line.
